# A Comparative Taxonomy of Parallel Algorithms for RNA Secondary Structure Prediction

**DOI:** 10.4137/ebo.s4058

**Published:** 2010-04-09

**Authors:** Ra’ed M. Al-Khatib, Rosni Abdullah, Nur’Aini Abdul Rashid

**Affiliations:** The Parallel and Distributed Computing Center (PDCC), School of Computer Sciences, University Sains Malaysia, 11800 Penang, Malaysia. Email: rmaak.cod09@student.usm.my

**Keywords:** RNA secondary structure, dynamic programming (DP), pseudoknot, free energy minimization, FPGA, GPU

## Abstract

RNA molecules have been discovered playing crucial roles in numerous biological and medical procedures and processes. RNA structures determination have become a major problem in the biology context. Recently, computer scientists have empowered the biologists with RNA secondary structures that ease an understanding of the RNA functions and roles. Detecting RNA secondary structure is an NP-hard problem, especially in pseudoknotted RNA structures. The detection process is also time-consuming; as a result, an alternative approach such as using parallel architectures is a desirable option. The main goal in this paper is to do an intensive investigation of parallel methods used in the literature to solve the demanding issues, related to the RNA secondary structure prediction methods. Then, we introduce a new taxonomy for the parallel RNA folding methods. Based on this proposed taxonomy, a systematic and scientific comparison is performed among these existing methods.

## Introduction

Bioinformatics is a new discipline resulting from the combination of two science fields: Computer Science, and Biology. This new scientific application has grown rapidly and nowadays it is becoming a cornerstone for each molecular biological study.^1^ It utilizes computer implementations and algorithms for collecting, accumulating, storing, analyzing, and integrating biological data and genetic macromolecules like deoxyribonucleic acid (DNA), ribonucleic acids (RNA), or proteins. The DNA has directions on how to build other components of cells, such as proteins and RNA molecules. The RNA is a type of nucleic acid that provides a mechanism to copy the genetic information of the DNA. There are two different types of RNA which are the Messenger RNA (mRNA) and the Transfer RNA (tRNA). They play important roles in the living cells and protein synthesis. Recently, researchers have found that they can use the RNA interference (RNA*i*) process^2^,^3^ for producing modern drugs. Mainly, this could be used in the therapeutics process of discovering antiviral drugs for difficult diseases like Cancer, AIDs, and Herpes.^4^,^5^

It is important to study the folding of RNA molecules to understand their roles and functionalities.^6^ Medical researchers and biologists can find different vital roles of RNA molecules by scrutinizing their RNA secondary structures. This will pave the way in front of biomedical researchers to utilize the RNA molecules in useful application like when there are used for productive treatments.^4^ The experimental methods that are commonly and broadly used for determining three-dimensional (3D) structures of RNA are listed as follows: X-ray crystallography and Nuclear Magnetic Resonance (NMR). In a biological context, these experimental methods are the most prominent accurate methods to determine the RNA tertiary structure, which is a 3D structure. But, both of these physical methods are time-consuming, expensive and computationally difficult to accomplish. To give the reasons, which make these purification techniques for determining the RNA 3D structures are tedious and difficult. In the rest of this section, we elaborated more about how both of these experimental methods are carried out by chemical or biological researchers.

**Figure 1.**
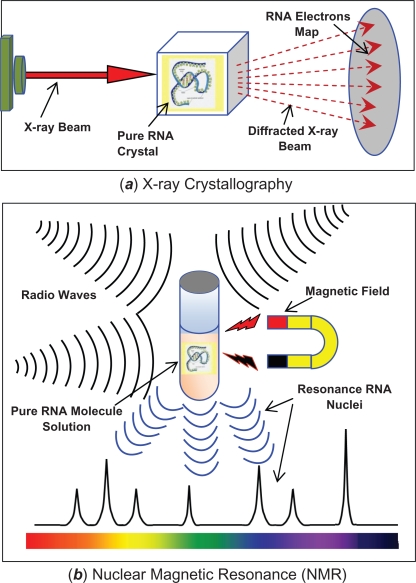
Experimental Methods of 3D RNA Structures Determination: (***a***) X-ray Crystallography sequence. (***b***) Nuclear Magnetic Resonance (NMR). (The idea adapted from^7^,^8^).

- *X-ray crystallography*. Primarily, it is the faster and popular purification experimental methods, to determine the RNA tertiary structures. The researchers fixed a pure crystal of single RNA molecule. They needed to obtain sufficient amounts of pure RNA crystal, a milligram of RNA quantity is necessary,^7^ which is complex and non-trivial to acquire. Next, the biologist will bombard this RNA crystal with X-ray, which is depictured in Figure 1 (a) as an adapted from.^8^ The X-ray beams will collide with the fixed and stable RNA crystal and will go through it. The X-ray will diffract whilst colliding with the electrons that are situated around the nuclei of RNA molecule. Thus, the result is the map for the electrons of the RNA molecule, which gives the nearest accurate form of the folding RNA molecule.
- *Nuclear Magnetic Resonance* (NMR). In this NMR experimental method,^7^ the charged RNA molecule is fixed via magnetizing the RNA molecule, as shown in Figure 1 (b). The magnetic field will stabilize the RNA sample. The phosphate groups within the backbone of the RNA have a negative charge which causes the solution of the RNA molecule to be charged.^9^ Then, the fixed molecule will be bombarded by using radio waves. This bombarding process will cause the resonation of the RNA nuclei. The 3D structure of the RNA molecule will be constructed from the resonating of these bombarded nuclei. However, this bombarding process needs to be performed from thousands of different angles. Therefore, this makes the NMR physical method incredibly time-consuming, costly and tedious.

Due to these constraints and difficulties from the experimental physical sides, computational methods from computer scientists and bioinformatic researchers have become more demanding to do the RNA structure prediction process. Predicting the RNA 3D structure from the primary sequence is difficult to accomplish. Hence, the bioinformatic researchers first detect and predict the RNA secondary structure from a given RNA primary sequence. Then, the result of this RNA secondary prediction process will assist the biologists to determine the RNA tertiary structure.

Thus, solving the RNA secondary structure problem is becoming a main issue among bioinformatic researchers.^10^ Recent approaches and current work concentrate on applying the parallel techniques to the previous RNA computational algorithms. In this paper, the works of the researchers explore the state-of-the-art of parallel techniques were proposed for solving RNA structural problems.

The earliest prediction methods for solving the RNA secondary structure problems were presented by Waterman and Smith^11^ and Nussinov et al.^12^ These computational algorithms proposed two different methods for the RNA/DNA folding structure, which require *O*(*n^3^*) time complexity. Later, several stable algorithms for the RNA secondary structures were proposed like Zuker’s algorithm.^13^ It was based on a thermodynamic energy minimization model. The execution time is still also *O*(*n^3^*), where *n* is the length of the RNA sequences, which are implemented in the *ViennaRNA package*.^14^

Basically, most computational algorithms, approaches and methods for solving the RNA secondary structures problems were restricted to the length of the bases, which were only a few hundreds of characters. Computational methods become much more desirable to solve traditional RNA cases and original RNA algorithms.^15^ Then subsequently, promising passageway simplifies these constraints and solves the bottleneck for the original RNA computational prediction algorithms, by implementing them on new parallelization approaches. Some of the more parallel popular approaches to reach this objective are to design them on the Field Programmable Gate-Array (FPGA), the Graphics Processing Unit (GPU), the multi-core or the cluster master-slave parallel architecture systems.^16^

## Schematic Study

We begin this comparative taxonomy study for the RNA parallel prediction methods by compromising and organizing this paper into three main research issues; (*i*) literature and background of the RNA predictions, (*ii*) RNA research models, (*iii*) the discussion and evaluation of existing methods with a comparative taxonomy. The main objective of this research is to present a comprehensive summary of the state-of-the-art researches on the RNA secondary structure predictions methods. Thus, we combined and joined among these all parts as follows:

- *The First part* is on the literature and background of the RNA prediction work. It explains the cause of the formation of folds and helices in each RNA molecule, in the chemical context. Next, the authors elucidated the RNA primary and the RNA secondary structure as a base step to clarify the RNA problem domain and the research statement on the computer side.
- *The next part* was for the RNA research models and methods. The researchers presented the existing RNA methods including the sequential and the parallel structural predictions. Then, the researchers made a comparison among either of the group. Lastly, in the discussion and comparison section, the authors highlighted the results of this study. In the discussion, the researchers concluded with the most suitable efficient paradigm that could be used for parallelizing the RNA prediction algorithms. Also, the reasons that guided to choose this decision were given.
- Finally, there is the discussion and the evaluation of existing methods with a comparative taxonomy. The researchers proposed and described a general taxonomy for RNA parallel methods. By using this taxonomy, they applied a comparative process for the previous existing parallel RNA prediction methods. This process enabled the researchers in this field to distinguish among the parallel RNA prediction approaches. Future researchers will be able to choose the suitable parallel paradigm that will fit their interests. This selection will depend on the group of the RNA molecules that the bioinformatic researchers working on or needs.

*Roadmap of the study*: The following section highlights the elementary RNA chemical structure and the RNA (*Primary* and *Secondary*) structures. Next, the authors presented the fundamental concept for the RNA secondary structure problem statement. Then, they listed and investigated the sequential RNA prediction methods and categorized the RNA parallel techniques based on the parallelization hardware architecture which are listed as follows: (*i*) An individual RNA parallel algorithm^15^ on a multi-core Central Processing Unit (multi-core CPU). (*ii*) A one solitary RNA parallel method on the GPU.^16^ (*iii*) One work of the RNA parallel algorithm on the Beowulf cluster.^17^ (*iv*) Three different parallel RNA algorithms were implemented on the FPGA.^18^–^20^ To complete the categorization, the researchers explained a suggested parallel taxonomy for RNA folding algorithms. Then, they systematically applied this proposed taxonomy on the mentioned RNA parallel methods, comparatively. Next, they discussed and compared the results for the main parallel RNA methods. Finally, the authors gave some concluding remarks. Also, they introduced some recommendations for bioinformatics researchers. These hints could be used as a constructive future research in the RNA structural prediction domain.

## Background

The RNA molecules had been confirmed to be very resourceful materials by.^3^,^4^ The RNA molecules play different crucial functions and roles in living organisms and in many biological processes. Recently, it became clear that the RNA molecules play various roles, not just an intermediate in protein synthesis. But also, the latest researches are looking to utilize the RNA*i* process to discover new medications as a treatment for dangerous virus diseases.^4^,^21^

### RNA chemical structure

To understand the roles and functions of the RNA molecules, the biomedical engineers and researchers need to determine and scrutinize the RNA tertiary structures. As the first step to reach this goal, they ought to know the RNA molecules chemical structure and the motives that forced them to make these folds of 3D structures. Accordingly, the RNA was the common name for ribonucleic acids; they are made of long chains (single-stranded) of nucleotide units. There are three different components of the RNA nucleotides: the nitrogenous base, the sugar, and the phosphate group. While, the RNA backbone is made up of ribose five atom carbon-sugar counted from 1′ through 5′ and it is attached by two phosphate groups in 3′ and 5′, respectively. From the sugar group “*ribose*”, the RNA molecule acquired its nickname “Ribonucleic Acids”. The nitrogenous bases in the RNA group were made up of four different bases Adenine (A), Cytosine (C), Guanine (G), and Uracil (U). These bases attached to the five-carbon sugar in 1′ position and they give RNA molecules characteristic possessions and properties.

Veritably, the phosphate groups in the backbone of the RNA have a negative charge, which makes the RNA a charged molecule.^9^ Due to this, the charged RNA molecule inside the living cells is not stable. Thus to gain more stability, some parts of the single-stranded RNA fold back on itself forming double helices. This RNA folding process makes the determination methods intricate and not easy to determine the RNA 3D structures. The details about the RNA molecules and its chemical structure was explained in.^22^

### The RNA primary and secondary structure

Both the phosphate groups are attached to the 3′ and 5′ positions from ribose sugar in the backbone of RNA molecules. Due to this fact and based on the convention and general agreement among biologists, the RNA primary structure is a string series of bases reported from the 5′ end to the 3′ end, as shown in Figure 3(a).

The RNA secondary structure derives from the pairing up of these four nucleotides according to the rules of *W*atson-Crick and *W*obble “*WW*”: *W*atson-Crick base pairs (G ≡ C) and (A = U) and a *W*obble base pair (G-U). By applying these rules, the single-stranded RNA secondary structure forms two large groups: [Stem-loops and Pseudoknots (PK)], as shown in Figure 3 (b and d).

## Problem Statement

**Figure 2.**
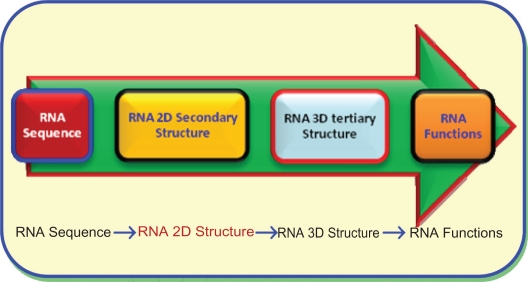
The main systematic chain steps of RNA research study.

Recently, RNA molecules have been confirmed to be very resourceful materials in the medical process and biological systems.^23^ Biologists can determine the RNA tertiary structure from its secondary structure. The biologists need this 3D structure of the RNA molecule to derive its function and essential role. Thus, the bioinformatic researchers introduced computational prediction methods, which can predict the RNA secondary structures from a given RNA primary sequence. In Figure 2, the authors depictured and explained the vital crucial position of the RNA secondary structure in the chain of the RNA research. Also, this figure showed the flow chain of the RNA research study, consecutively. Moreover, it confirmed that the computational secondary structure prediction processes is a required preliminarily step for determining the RNA 3D structure.

**Figure 3.**
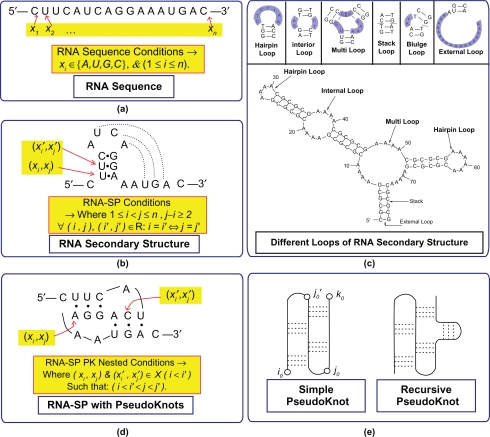
RNA Structures:- (**a**) RNA sequence. (**b**) RNA secondary structure. (**c**) RNA Stem-Loops Structure. (**d**) RNA PseudoKnots. (**e**) PseudoKnots Types [Simple and Recursive], some parts adapted from.^10^,^15^,^25^

The RNA folding recognition methods attempt to predict an accurate and more stable RNA folding structure based on the Minimum Free Energy (MFE) models. As shown in Figure 3 (d), the nature of some types of the RNA structure forms the pseudoknots, in some parts. The RNA molecules with the pseudoknots structure make the calculation process of the RNA secondary structure prediction algorithms complex. These complexities of the RNA prediction algorithms conform and confirm the execution time and memory storage space, computationally.

The components of the RNA involved in understanding the RNA functions, which are extracted from,^24^ and can be presented as follows:

- RNA primary sequence structure is a string of *n* characters, x*_i_* = *x*_1_*x*_2_*…x_n_* where x*_i_*∈ {*A or a, C or c, G or g, U or u*} the four bases in uppercases or lowercases letters, as well 1≤*i*≤*n*, as seen in the Figure 3 (a).
- A single-stranded RNA secondary structure is a list of base-pairs which can be viewed as a set of, X, forms on acceptable base pairs (*x_i_, x_j_*). These pair of letters is called a complementary base pair, according to the “*WW* ” rules, in Figure 3 (b):
  - These “*WW*” rules are: (*x_i_, x_j_*) = (*a,u*) or (*A,U*), (*x_i_, x_j_*) = (*g, c*) or (*G, C*) for the Watson-Crick rule. Also, (*x_i_, x_j_*) = (*g, u*) or (*G, U*) along with the Wobble rule later.
  - In addition, these base pairs (*x_i_, x_j_*) for RNA secondary structure where that:
    - It should be at *first*, 1≤*i<j*≤*n*.
    - *Second*, *j*–*i>t* where *t* is a small constant, i.e. *j–i*≥2.
- For all base pairs (*x_i_, x_j_*) and (*x_i_*′*, x_j_*′) in X, *i = i*′, if and only if *j = j*′, (such that ∀ (*i, j*), (*i*′*,j*′)∈ R: *i = i*′ ⇔ *j = j’*) as depictured in Figure 3 (b).
- Thus, namely two bases that form the canonical pair must be located at different locations. While, the RNA sequence does not fold back on itself too sharply. Also, each base can be paired and combined at most only with another base. Restrictedly, the implementers allowed just *“WW*” the canonical RNA base pairs rules^26^: {(A,U), (C,G), (G,U)}. The RNA secondary structures without pseudoknots folding in different kinds of loops,^15^ which are: Hairpin Loops, Internal Loops, Multiloops, Stacks, Bulges and External Loops, as depicted in Figure 3 (c).
- *The RNA secondary structure with pseudoknots*: this is defined as, X, if and only if the base pairs exist in nested condition (*x_i_, x_j_*), (*x_i_*′*, x_j_*′)∈ X (*i<i*′) such that *i*<*i*′<*j*<*j*′, as seen in the Figure 3 (d). Whereas the RNA secondary structures with pseudoknots coming in two main groups of pseudoknotted RNA^10^: *simple* or *recursive,* those depictured in Figure 3 (e). Thus, a given RNA sequence X, can fold with a maximum number of base pairs and in an exponential number of possible structures.

### Thermodynamic algorithm for RNA prediction

From the energy stability point of view, the phosphate groups in the backbone of RNA molecule have a negative charge. Thus, the RNA molecules inside the living cells are not stable.^9^,^27^ They will fold back on themselves to reach more stability.

Then, the main goal of the RNA secondary structure prediction computational method is to arrive at more stable equilibrium of the RNA folding form, based on the free-energy model. Hence, to calculate the RNA free energy stabilities, it is necessary to predict RNA secondary structure by calculating the MFE, which is named as an optimal RNA structure.^28^

The summation of energies for all loops is the energy of RNA secondary structure (Eq. 1), which extracted with condensation from.^18^,^28^,^29^ The empirical calculations explained and confirmed that over 99% from the execution time for the RNA prediction algorithm is in computing the MFE.^20^

(1) $$\begin{array}{c} \text{Total}\;\text{MFE}\;\text{for}\;\text{RNA}\;=\;\sum \text{of}\;\text{RNA}\;\text{Loops}\;\text{Energies}\\ \left(\text{At}\;\text{fixed}\;\text{temperature}\;+\;\text{ionic}\;\text{concentration}\right) \end{array}$$

In reality, to the best knowledge of the authors, thermodynamic prediction approach for calculating the energy of the optimization RNA, had been expressed and introduced for the most time by Lyngso *et al* in.^28^ They were used a four different arrays to hold and include the MFE lookup tables inside the shared cache memory during the execution time of the prediction algorithm.

These MFE calculative motifs complicate the general pseudoknotted RNA secondary structure prediction algorithms. The pseudoknots type turns the RNA prediction algorithms to be *NP-Complete* Problem.^10^,^30^ In addition, the algorithms for solving the pseudoknotted RNA secondary structure problem, need to allow energy functions to operate and run in the worst case polynomial time. In fact, two researchers Akutsu in^10^ and Lyngso and Pedersen in^30^ proved that finding the pseudoknotted RNA structure with the MFE is the *NP-hard* problem, particularly by applying the standard nearest-neighbour energy function.

Consequently, researchers of pseudoknotted RNAs faced with three problems: *First*, the RNA secondary structure prediction with the pseudoknots is computationally intricate and difficult to carry out.^30^ Considering the execution time and the memory space complexities, made the problem an *NP*-complex problem.^30^ Besides that, most professional algorithms exist only for partial classes of pseudoknots.^10^,^25^,^31^,^32^ Namely there were restricted in a subclass and not for all classes of pseudoknotted RNA.^33^ *Second*, almost majority of RNAs computational methods have analyzed nested “Stem-Loops” RNA secondary structure prediction,^34^ either by neglecting the RNA pseudoknots for simplicity, or not being aware of the existence of the pseudoknotted RNA types. *Lastly*, most of the existing methods for RNA structure prediction are not acceptable at levels of accuracy, reliability and robustness.^33^

## Existing Sequential Methods

Basically, one of the important tasks in front of bioinformatics and computer application researchers is the RNA secondary structure prediction dilemma. From a biological point of view, there are various complexities and constraints that are faced the biomedical researchers in experimental methods to determine the RNA tertiary structures.^7^ Recently, many computational efforts have been presented on the computer side, to detect the RNA secondary structure from the primary sequence. The predictive approaches have been introduced to solve the related issues in the RNA structural detection field as follows: (*i*) Energy thermodynamic models to predict RNA secondary structures; i.e. Mfold,^13^ RNAfold^14^ and RNAalifold.^35^ (*ii*) Comparative analysis methods to predict the RNA secondary structures from multiple homologous sequence alignment.^36^ (*iii*) Stochastic Context-Free Grammar methods (SCFS) are a comparative sequence analysis for prediction consensus of the RNA secondary structures from multiple sequence alignment.^37^

The original existing methods that have been proposed in solving the RNA secondary structures are divided into two major groups. The first group predicts the *non-pseudoknotted* RNA secondary structures “*Stem-Loop of RNA*”, while the other group is solving the RNA secondary structure prediction problem including pseudoknots type “*RNA with Pseudoknots*”.

**Figure 4:**
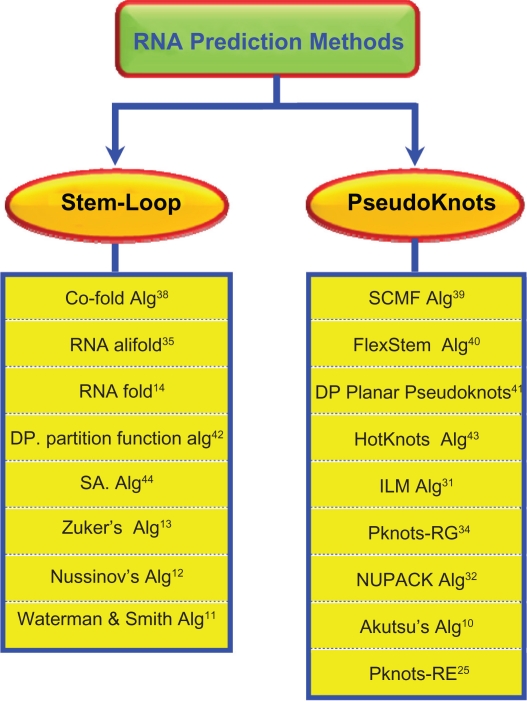
Schematic diagram of RNA structural prediction methods.

Then, the researchers in this paper classified these sequential RNA prediction methods in a schematic diagram, as shown in Figure 4. Next, the researchers converged these RNA prediction methods, as shown in Table 1, which was adapted and extracted from.^22^ This table included the most well-known existing RNA sequential prediction methods and approaches that have been produced lately to predict RNA secondary structures.

## Parallel Methods and Schemes

The experimental methods are completely accurate for determining the RNA 3D folding structures. But due to their time consuming and expensive nature, many computational approaches have been proposed to predict the RNA secondary structure, which includes: (*i*) RNA prediction methods based on a thermodynamic energy minimization model. (*ii*) RNA structural comparative approaches from multiple homologous sequences. (*iii*) A comparative prediction consensus of the RNA structures by using SCFG methods. (*iv*) The Genetic Algorithm (GA) for predicting RNA structures.^18^

Lately, many different parallel methods were introduced, in order to face the computational complexities of the RNA secondary structure prediction problem such as: (*i*) The scalable program using the parallel multi-core.^15^ (*ii*) An implementing computer algorithms to run on the graphics hardware like GPU.^16^ (*iii*) A parallel implementation on the Beowulf cluster by using Message Passing Interface (MPI) library.^17^ (*iv*) The fine-grained hardware implemented on FPGA.^18^–^20^ In the remaining parts of this section, the authors discussed the existing parallel methods for predicting the RNA structure from a given primary sequence.

### Multi-core RNA parallel algorithm

Recently, the mainly accepted and accurate approaches for predicting the RNA secondary structures sequentially are *Mfold*^29^,^46^ and *RNAfold*.^14^ These two sequential approaches require the *O*(*n*^4^) execution time steps and the *O*(*n*^2^) spatial storage complexities. Having these complexities, the prediction of the large RNA sequences would not be feasible, particularly in the sequential implementation. Lately, one RNA research presented a parallel and scalable design “*GTfold*” in.^15^ GTfold was implemented on the multi-core CPU for solving the RNA secondary structure problem. This parallel RNA prediction method integrated the *Mfold* and *RNAfold* algorithms together, in the parallel blueprint. This proposed re-implemented parallel method “GTfold”^15^ obtained more accuracy in predicting RNA secondary structure. Also, it could compute the larger RNA sequences.

In fact, the value and the significance of the *GTfold* is in predicting the accuracy of the large RNA sequences. This accurate result when compared to many other existing RNA approaches such as the *Mfold*,^29^,^46^ and the *RNAfold*.^14^ Also, Mathuriya et al in^15^ made an optimal improvements by reducing the time complexity from *O*(*n*^4^) to *O*(*n*^3^). This improvement was calculated in computing the internal loop in the RNA fold, by improving the Internal Loop Speedup Algorithm “*ILSA*”. Basically, this *ILSA* enhancement enabled the execution steps of the *GTfold* algorithm, to run in a shorter time.

Therefore, the *ILSA* enhancement gave the opportunity for the *GTfold* to predict *Homo sapiens 23S* ribosomal with 5,184 nucleotides of the RNA sequence only in minutes compared to the nine hours before.^15^ Furthermore, the *GTfold* calculated the HIV-1 viral RNA genomes in 84 seconds (two months before^47^); HIV-1 includes 9,781 bases of RNA nucleotides. The *GTfold* was a parallel implementation of the RNA secondary structure prediction in the 16-core dual CPU symmetric multiprocessor system on an IBM P5-570 server machine. The *GTfold* algorithm achieved a good execution time one to two times (a factor 1.6× speed-up time). This algorithm has shown enhancements in both efficiency and performance when compared to the existing sequential algorithms,^14^ in the large sequences.

### Parallel algorithm on GPU

The Graphics Processing Units (*GPUs*) started like a specific processor for accelerating and manipulating 3D computer graphical operations and games. Fortunately, due to the GPU’s highly parallel structure, extraordinary powerful and common function computing engines, this technique open a promising way in the parallel bio-computing sector. Recently, the General Purpose of GPUs (GPGPU) also has been growing very fast. The GPUs are going to become the cornerstone for the high computational complexity algorithms,^16^ like the pseudokotted RNA structural prediction methods, which was proved as the *NP-hard* problem.^10^,^30^

Basically, the researchers utilized the latest modern GPUs to speed-up the algorithms in solving RNA secondary structure problems. Rizk and Lavenier in^16^ explored a new implementation for the previous function, which was used in solving RNA prediction problems. The researchers re-implemented the *hybrid-ss-min*^48^ function on GPU. This function was used in the Unafold package to compute the MFE of the RNA folding problem. Also, the original RNA algorithm “Unafold” has a time complexity of *O*(*n*^3^). Consequently, the new parallel design^16^ on modern GPUs hardware fulfilled more accelerated speed-up time of up to ×17. These results was comparing with the same function which run on a system with a single CPU sequentially.

The significance of the research results in,^16^ is to obtain faster execution time in the RNA secondary prediction algorithm. Utilizing the GPU design, reduced the execution time and the computation complexities without any extra cost. This improves the results when compared with the other competitive RNA prediction methods like the Multi-core, the clusters or the multiprocessors systems.

### Parallel framework on cluster

The computer hardware Beowulf cluster showed some strong parallel features, based on the master-slave paradigm.^49^ Many researchers exploited this parallel architecture to compute the traditional RNA secondary structure detection methods. The parallel implementation on the Beowulf cluster was utilized for re-implementing the original RNA prediction methods.^17^ This work pointed out that there were good results with a higher accuracy and a faster execution time in the RNA structural algorithms, comparing to the original RNA detection methods.^25^,^34^ The pseudoknotted RNA complicated structure algorithms could also benefit from this parallel design. Despite those RNA pseudoknots structures were always recognized in most of the RNA molecules and they were known among RNA researchers.^24^ But, due to that the pseudoknotted RNA molecules are computationally demanded nature. Thus, the RNA pseudoknots types mostly ignored from many RNA prediction methods. Namely, some researchers compute their RNA prediction methods without pseudoknots to get more simplicity.^34^ These perdition methods to solve RNA secondary structures would be inaccurate when they neglected pseudoknotted types.

A prominent work has opened a new epoch in pseudoknotted RNA secondary structure prediction research. The *CompPknots*^17^ integrated two main pseudoknotted RNA secondary structure prediction methods.^25^,^34^ The researchers implemented a parallel master–slave framework between two existing RNA methods. These two existing pseudoknotted RNA prediction methods are:

- *Pknots-RE*^25^: an optimal method in solving pseudoknotted RNA secondary structures. It was based on applying the standard RNA thermodynamic stability of pseudoknots type. The *Pknots-RE* method requires *O*(*n*^6^) in time and *O*(*n*^4^) memory space, where *n* is the length of the RNA sequences.
- *Pknots-RG*^34^: It was the latest improvement on time and space complexities for the same algorithm, which was used before in the *Pknots-RE*. The authors of *Pknots-RG* utilized the MFE model, to achieve better performance on the complexity, when comparing with the *Pknots-RE*.^25^ This new version is the *Pknotes-RG*, which enhanced the execution time complexity from *O*(*n*^6^) to *O*(*n*^4^). Also, *Pknots-RG* improved the storage complexity to *O*(*n*^2^) from *O*(*n*^4^) comparing with the *Pknots-RE* method.

The main contribution of the *CompPknots*,^17^ was to apply a parallel calculation in bioinformatics for the pseudoknotted RNA structural methods. The authors used the MPI library with the combination of these two previous pseudoknotted RNA algorithms^25^,^34^ in a parallelization design. The new paradigm enabled the researchers to predict larger RNA sequences. While, the two previous methods^25^,^34^ were not able to act as efficient as the improved parallel one.^17^ The *CompPknots* parallel design was implemented on the Beowulf cluster based on the master-slave architecture. This parallel design obtained the good results in the pseudoknotted RNA secondary structure detection with the higher accurate prediction. Also, the method ran in a shorter execution time. Moreover, the authors in^17^ introduced a new automatic comparison approach, which allowed the end users to compare their final results directly with previous ones. This automatically comparison process takes a shorter time than the traditional manual methods that they were using tools such as the Pseudoviewer3.^50^

### Parallel on FPGA co-processors

Different researches found and confirmed that the traditional RNA prediction methods can be put into operation by using fine-grained hardware implemented on the FPGA. Recently, the bioinformatic researchers found that the modern computers, parallel or multi-core, do not show a greater than 50% parallel usefulness and efficiency.^18^ But the researchers achieved better accelerations when using the FPGA co-processors, which grows to be a hopeful approach for the main RNA prediction algorithms like RNAalifold,^18^ Nussinov’s algorithm^19^ and Zuker’s algorithm.^20^

#### (i) Fine-grained parallel implementation on the FPGA for the RNAalifold method

The most popular computational approach for RNA secondary structures based on using the MFE-model is the *RNAalifold* folding method, which was presented by Hofacker et al in.^35^ The *RNAalifold* was used for computing the consensus of RNA structures and it had been implemented as an extension of *Zuker’s* algorithm.^13^ Also, the *RNAalifold* algorithm was considered the thermodynamic energy minimization stability with an average energy matrix and sequence co-variation score matrix together. The *RNAalifold* approach has the worst case execution time and storage space complexities are respectively *O*(*m* × *n*^4^ + *n*^3^) and *O*(*n*^2^), as *n* is the length of RNA sequence and *m* is the number of RNA sequences in the alignment. Due to these strong reasons Xia et al in^18^ adopted the *RNAalifold* algorithm and re-implemented it on FPGA chips.

The researchers in^18^ supplied an innovative accelerated approach for the *RNAalifold* algorithm. They proposed a systolic array structure with one master Processing Element (PE) and many slave PEs for fine-grained hardware implementation on the FPGA. Their goals from this re-implementation, was to parallelize the original *RNAalifold* algorithm. The master PE loads the energy matrices from the outer memory “*DRAM*”, while the other slave PEs remain waiting to take data from the master PE. The execution time of the parallel prediction algorithm was grown to be more than 12× on one FPGA with 16PEs. This results compared with the previous results for *ViennaRNA-1.6.5* software,^51^ the original *RNAalifold* algorithm.

#### (ii) Fine-grained parallelization of Nussinov’s algorithm implemented on FPGA

Many RNA folding methods utilized empirical models for predicting the RNA secondary structures based on the MFE estimation by using the Dynamic Programming (DP) algorithm. Jacob et al in^19^ pointed out that in all these empirical models, algorithms for predicting RNA structures are just DP recurrences from the original Nussinov’s algorithm.^12^ There are two features that make this algorithm computationally more applicable for predicting RNA secondary structures among the other prediction methods; Firstly, Nussinov’s algorithm used the length of RNA sequence as a proxy for the MFE and it computed the most maximum of RNA base pairs. Secondly, the original Nussinov^12^ one runs in *O*(*n*^3^) time and requires *O*(*n*^2^) storage space complexities.

Jacob et al in^19^ adapted one of the most recent parallel FPGA architectures (*Virtex-II* 6000 FPGA) for implementing the normal Nussinov’s algorithm. Also, the researchers calculated and built it on two classic two-dimensional (2D) systolic arrays to achieve optimal string parenthesization and to deal with the maximum length of RNA primary sequence. Hence, Jacob et al^19^ implemented this design of accelerating the original Nussinov RNA structural prediction method by utilizing the 2D systolic arrays on FPGA implementation. This parallel design obtained good throughput results on the *Virtex-II* 6000 FPGA. This output was compared with the same implemented method on the modern *x*86 CPU. The results obtained better factor up to 39× speed-ups in execution time.

#### (iii) Parallel fine-grained implementation for the Zuker algorithm on FPGA

The most well-liked and admired computational approach for the RNA secondary structure based on using the MFE is the Zuker’s algorithm.^13^ It was confirmed to be the most stable RNA secondary structure prediction method based on calculating MFE. The Zuker’s algorithm runs in *O*(*n*^4^) execution time and resides in the *O*(*n*^2^) storage memory requirements complexities. Recently, different developments and many optimal algorithms have been derived to simplify and reduce the Zuker’s algorithm computational complexities. Also, most biology researchers have proved that, the RNA molecule existing in large sequences is more than what was expected before. In other words, biological experiments have shown that RNA molecules fold in thousands of bases.^15^ Also, new discoveries make a huge increase of the RNA data sources; the data in GenBank almost doubling every year.^52^ The original polynomial time complexity of the Zuker’s algorithm is *O*(*n*^4^). This complexity could not be able to deal with these sophisticated instances introduced earlier, in a sequential manner.

The promising way to make Zuker’s algorithm tolerable and balanced in calculation is a parallel structural design implementation. Recently, Dou et al in^20^ proposed an innovative parallel design for accelerating Zuker’s algorithm. The investigators in^20^ utilized and built their techniques by dividing the Zuker algorithm matrix in the usual mode. Then, they submitted and distributed the subtasks as a multithreading procedure that can be calculated independently. This parallel scheme was implemented on the FPGA fine-grained hardware by proposing one master PE and multiple slave PEs with systolic array structure. In addition, the RNA researchers in^20^ projected new methods for reducing the RNA energy lookup table size by 85%. These lookup tables should be loaded in the memory, as the RNA prediction algorithm requires to use them. Consequently, they implemented their parallel algorithm on 16 PEs on FPGA co-processors. The experimental results of this parallel scheme explained enhancement up to factor of 14× speed-up time comparing with the *ViennaPackage*.^51^

## RNA Parallel Taxonomy

### Parallel taxonomy of RNA folding

**Figure 5:**
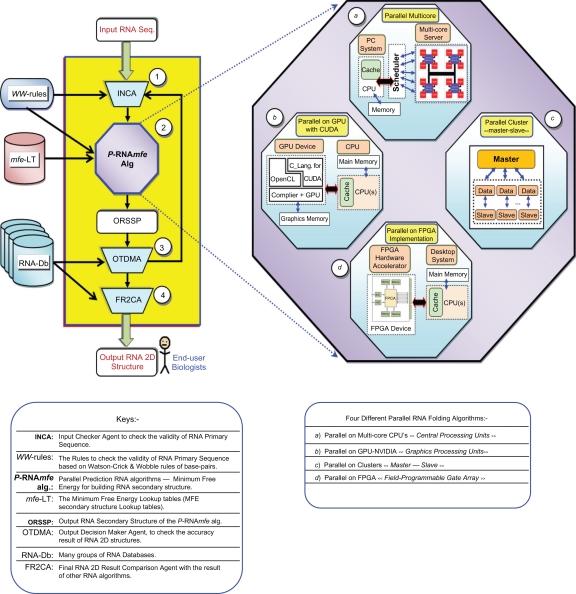
Parallel taxonomy of RNA folding algorithms. (**1**) INCA: Agent to check the validity of input RNA primary sequence. (**2**) *P*-RNA*mfe* Alg: Parallel RNA secondary structure prediction algorithm based on MFE. It zooms out in [**a**, **b**, **c** or **d**]. (**3**) OTDMA: Agent to compare the first result with existing online RNA databases. (**4**) FR2CA: Agent to measure the performance of the RNA structural prediction method with the standard benchmarks.

In particular, the most popular RNA secondary structure prediction methods are the dynamic programming algorithms based on the MFE models. Herein, we proposed and described a general taxonomy to apply on the existing RNA parallel methods. This proposed taxonomy for the RNA parallel secondary structure methods is using four main phases. Three of them working as a different agents and the fourth one is the main RNA prediction algorithm, as shown in Figure 5:

- The Input Checker Agent (INCA) receives the RNA primary sequence from the input device or reads it from a text file. Subsequently, the INCA checks the validity of this RNA by applying the valid nucleotides RNA bases in Watson-Wobble rules (*WW*-rules).^10^,^24^
- INCA transfers the valid RNA sequence to the parallel prediction RNA MFE algorithm (*P*-RNA*mfe*), which starts calculating the RNA secondary structure by using *WW*-rules to combine the valid RNA base-pairs. The *P*-RNA*mfe* algorithm simultaneously predicts the optimal and most stable RNA secondary structure by using the MFE lookup tables (*mfe*-LT).^15^,^28^ These *mfe*-LTs are loaded to the shared cache memory by loader just before the *P*-RNA*mfe* algorithm start calculating and predicting the RNA secondary structures. Essentially, based on this investigation, there are four parallel techniques was used to re-implement the previous RNA prediction methods. These four popular parallel RNA prediction methods were illustrated by zooming in the “*P*-RNA*mfe*”, as shown in Figure 5 (a, b, c and d). A brief explanation of these four implementation parallel methods are as follows:
  - *First*, a parallel design to harness the power of the multi-core CPUs for predicting the RNA secondary structures. The *GTfold* is a parallel multi-core algorithm.^15^ It computed a larger RNA sequences, as shown in Figure 5 (a).
  - *Second*, a parallel paradigm on the latest modern GPU-NVIDIA cards. This parallel design was utilizing the CUDA programming code in the C-language environment. This new scheme was used in^16^ to accelerate the execution time for the previous RNA folding method.^48^ This design was explained in Figure 5 (b).
  - Another parallel design is a combination of two existing pseudoknotted RNA secondary structures prediction methods.^25^,^34^ This design^17^ used a parallel master-slave techniques based on the Beowulf cluster/hardware, as depictured in Figure 5 (c).
  - The last parallel scheme elaborated the 2D systolic array parallel by using FPGA in fine-grained hardware. This parallel paradigm was illustrated in Figure 5 (d). Actually, there were three various parallel RNA detection methods.^18^–^20^ These original RNA prediction methods were lately re-implemented on FPGA.
- The Output Decision Maker Agent (OTDMA) checks the accuracy of the initial Output RNA Secondary Structure Production (ORSSP). It performs this checkable process by testing and comparing the first output “ORSSP” with the diverse known RNA structures (as a standard benchmark for testing and evaluating the RNA prediction method) in the RNA Databases (RNA-Db).^53^–^57^ If the OTDMA finds the quality of the new ORSSP is poor or low, it feeds the RNA sequence back to the INCA to re-start another round of detection with new and more intelligent constraints. Or else, the OTDMA finds the accuracy of the output RNA structure is high, it transfers the new ORSSP to the last agent, which called Final Result 2D Comparison Agent (FR2CA).
- The FR2CA compares the final RNA secondary structure result with the known and existing structures in RNA-Db, to report the accuracy scale of predicted RNA.^58^ In addition, the FR2CA agent measures the execution time and space complexities for the RNA prediction algorithm.

### Evaluating existing RNA algorithms using comparative taxonomy

By using this proposed comparative taxonomy, as a classified comparison procedure for the existing parallel RNA prediction methods. The authors found that some of these RNA parallel prediction methods ran through all the comparative taxonomy phases. While, the other RNA prediction methods went through some steps of this proposed comparative taxonomy.

From the methodological point of view, the researchers explained and investigated a comparison procedure among these RNA parallel methods according to the proposed taxonomy in Table 2. The selected group of the previous RNA algorithms focuses on the parallel implementation of the existing RNA secondary structure methods.

## Discussion and Comparison

Scores of RNA researchers have introduced several evolutionary parallel blueprints for solving the RNA secondary structure problem. In this paper, the researchers discussed and compared these parallel RNA methods, as shown in Table 3. They have discussed in^22^ an intensive RNA detection algorithms, as a first phase of the RNA secondary research in a Bioinformatics domain. The authors classified and compared the RNA approaches in two main groups and they presented the results in the two tables. In this study, the researchers extracted the well-known RNA sequential prediction methods in Table 1.

Then, in this paper, the authors focused their analysis on discussing and comparing the latest parallel RNA prediction efforts that have been pioneered in the RNA secondary structure folding domain. Consequently, from the comparison of the parallel RNA methods in Table 3, it could be noticed that only one research^17^ presented a parallel design for the RNA pseudoknots type. While the others, proposed parallel designs for the RNA detection on stem-loops types. The comparison had also shown that the latest parallel algorithm^16^ applied on the GPU, by utilizing the power of the NVIDIA card with the CUDA program. This method was implemented on the GPU to solve the RNA secondary structure problems. The high accessibility of the GPU card in the contemporary machines and the provided features for the developer to utilize GPU using high level languages like a C language environment; are the important motivations for the RNA research community to exploit the CUDA on the GPU.

Finally, the authors concluded that, implementing the RNA secondary structure prediction methods on parallel architectures has several significant benefits. The main advantage is reduced complexity, in both time and memory storage, comparing with the original RNA structure prediction algorithms. Also, the parallel RNA algorithms provided accurate results with better performance.

## Conclusion

In this paper for the solving RNA secondary structure prediction problem, the authors presented the state-of-the-art of the RNA parallel methods. They introduced an intensive investigation on exhaustive up to date parallel RNA secondary structure prediction algorithms. Indeed, recently various methods and techniques for predicting RNA secondary structures have emerged. Most of these computational methods have faced with some complexities, from sequential implementation viewpoint. Therefore, re-implementing the existing RNA prediction methods by using parallelization architectures would result and contribute in solving the faced difficulties. The study concluded and showed that all of RNA parallel methods obtained better results, when compared to the sequential methods in terms of accuracy in one side and time/space complexities, in another side.

This research study was comprised in three trends: (*i*) RNA in biological context. (*ii*) RNA computational prediction methods. (*iii*) A comparative taxonomy of RNA parallel methods. In the first part, the researchers explained the experimental method’s difficulties that the biologists are facing, in determination of RNA 3D structures. Exploratively, the researchers concluded the reasons that force and allow the RNA molecule to fold and pair back on itself forming a double helices, in a chemical and biological context. Secondly, in the methods and research findings, the authors listed the sequential RNA secondary structure methods, in a schematic classification diagram. Then, they compared their contributions and complexities. Also, the authors investigated comprehensively the state-of-the-art of the RNA parallel prediction methods.

In addition, the researchers performed a scientific comparison among these enhanced RNA parallel methods with the previous existing methods. In the third part, the researchers proposed a new parallel taxonomy. Then, they applied the existing parallel methods using this taxonomy. Lastly, the researchers conducted a comparison procedure to evaluate these RNA parallel methods based on the proposed taxonomy, in terms of the taxonomy steps.

Consequently, this study of the RNA existing parallel methods proved that the parallelization performance of the algorithm is proportional to the method of the parallelization itself. Particularly, the comparison showed that the proposed RNA methods utilizing GPU capabilities result more promising outputs. Besides that, from the implementation point of view, the available open source Application Programming Interface (API) in a high level language in C environment could be considered as a positive point. These parallelizing RNA secondary structure prediction methods, showed a promising area for future RNA studies and for computational RNA bioinformatic researches.

## Figures and Tables

**Table 1:** Existing sequential methods for RNA secondary structure prediction.

**No.**	**RNA prediction method**	**Reference**	**Method complexities**		**Major contribution**	**RNA types**
			**Execution time**	**Space requirement**		
1.	SCMF Alg.	Jens and Andrew^39^	*O*(*m n*^2^)	*O*(*n*^2^)	A near optimal algorithm to predict RNA secondary structure with pseudoknots. Where *n*: is the RNA sequence length. And *m*: is the number of iteration steps with the *n* bases.	Pseudokots
2.	FlexStem Alg.	Chen et al^40^	*O*(*n*^4^)	*O*(*n*^2^)	A prediction algorithm named for RNA secondary structures, it adapted a comprehensive energy models for complex pseudoknots type. Where *n*: is the RNA sequence length.	Pseudokots
3.	Co-fold Alg.	Ziv-Ukelson et al^38^	*O*(*n*^4^*ζ* (*n*))	–	An optimal alignment alg. to predict RNA secondary structures based on Sankoff’s Alg.^44^ Where *n*: is the RNA sequence length. And ζ(*n*) can converge to O(*n*).	Stem-Loops
4.	DP Planar Pseudoknots	Hengwu et al^41^	*O*(*n*^4^)	*O*(*n*^3^)	A DP algorithm to predict RNA secondary structures with arbitrary planar and simple non-planar pseudoknots type by using MFE model. Where *n*: is the RNA sequence length.	Pseudokots
5.	HotKnots Alg.	Ren et al^43^	*O*(*n*^4^)	*O*(*n*^2^)	A heuristic algorithm to predict pseudoknotted RNA based on MFE. Where *n*: is the RNA sequence length.	Pseudokots
6.	ILM Alg.	Ruan et al^31^	*O*(*n*^4^)	*O*(*n*^2^)	A heuristic algorithm for predicting pseudoknotted RNA bosed on MFE or comparative or both. Where *n*: is the RNA sequence length.	Pseudokots
7.	Pknots-RG	Reeder et al^34^	*O*(*n*^4^)	*O*(*n*^2^)	A DP algorithm to predict optimal RNA secondary structures by using MFE model. Where *n*: is the RNA sequence length.	Pseudokots
8.	NUPACK Alg.	Dirks and Pierce^32^	*O*(*n*^5^)	*O*(*n*^4^)	A DP algorithm to predict base-pairing probabilities of RNA with pseudoknots based on a partition function and MFE. Where *n*: is the RNA sequence length.	Pseudokots
9.	RNAalifold	Hofacker et al^35^	*O*(*m × n*^4^*+ n*^3^)	*O*(*n*^2^)	The algorithm computes the consensus RNA secondary structures from multiple alignments with modifying energy models. Where *n*: is the RNA sequence length. And *m*: is the number of sequences alignments.	Stem-Loops
10.	Akutsu’s Alg.	Akutsu^10^	*O*(*n*^4^)	*O*(*n*^3^) enhanced by^30^ to be *O*(*n*^2^)	A simple DP algorithm to predict RNA secondary structure with pseudoknots. Where *n*: is the RNA sequence length.	Pseudokots
11.	Pknots-RE	Rivas and Eddy^25^	*O*(*n*^6^)	*O*(*n*^4^)	An adaption of DP algorithm for predicting a tractable subclass of pseudoknotted RNA based on complex MFE model. Where *n*: is the RNA sequence length.	Pseudokots
12.	RNAfold	Hofacker et al^14^	*O*(*n*^3^)	*O*(*n*^2^)	An implementing of Zuker’s RNA prediction alg.^13^ based on MFE model with employing thermodynamic parameters of.^29^ Where *n*: is the RNA sequence length.	Stem-Loops
13.	DP. partition function alg.	McCaskill^42^	*O*(*n*^3^)	*O*(*n*^2^)	A DP algorithm used MFE model to predict the partition function of unpseudoknotted RNA. Where *n*: is the RNA sequence length.	Stem-Loops
14.	SA. Alg.	Sankoff^44^	*O*(*n*^6^)	*O*(*n*^4^)	A DP algorithm for RNA secondary structural alignment. Where *n*: is the RNA sequence length.	Stem-Loops
15.	Zuker’s Alg.	Zuker and Stiegler^13^	*O*(*n*^4^) optimized by^45^ to be *O*(*n*^3^)	*O*(*n*^2^)	An improved DP algorithm to predict RNA secondary structures from single sequence by computing MFE. It has been re-implemented by Mfold,^13^ RNAfold^14^ and RNAalifold.^35^ Where *n*: is the RNA sequence length.	Stem-Loops
16.	Nussinov’s Alg.	Nussinov et al^12^	*O*(*n*^3^)	*O*(*n*^2^)	A simplest DP algorithm computes RNA secondary structure based on MFE. Where *n*: is the RNA sequence length.	Stem-Loops
17.	Waterman and Smith Alg.	Waterman and Smith^11^	*O*(*n*^3^)	*O*(*n*^2^)	A simple DP algorithm for predicting RNA secondary structure without pseudoknots. Where *n*: is the RNA sequence length.	Stem-Loops

**Table 2:** The evaluating taxonomy for the parallel RNA secondary structure prediction approaches.

***Taxonomy Phase***						
**RNA Parallel Method**	***GTfold* RNA Alg.^15^*on Multi-core***	**Accelerated RNA Alg. on *GPU*^16^**	***compPknots*: RNA Alg.^17^*on Cluster* «*Master–Slave*»**	**Parallelize *RNAalifold* Alg. On FPGA^18^**	**Accelerating *Nussinov* Alg. on FPGA^19^**	**Parallel *Zuker* Alg. on FPGA^20^**
Check valid RNA Seq. by *INCA*	✓	✓	✓	✓	✓	✓
Applying canonical *WW-*rules	✓	✓	✓	✓	✓	✓
Using mfe-Lookup tables “*mfe*-LT”	✓	✓	✓	✓	✓	✓
Comparing the 1st output with existing RNA structure via *OTDMA*	✓	✓	Automatically comparing	comparing to ViennaRNA	Only with original *Nussinov*	comparing to ViennaRNA
Compare accuracy by *FR2CA*	mfold^29^,^46^ and RNAfold^14^	Unafold^48^	Pknots-RE^25^ and Pknots-RG^34^	RNAalifold^35^	Nussinov Alg.^12^	Zuker Alg.^13^
Types of RNA including prediction	Stem-loop RNA Structure	Stem-loop RNA Structure	Pseudoknotted RNA Structure	Stem-loop RNA Structure	Stem-loop RNA Structure	Stem-loop RNA Structure
Parallel Framework	Parallel Multicore and Scalable Program by using OpenMP	CUDA programming on GPU card	Master-slave paradigm using MPICH library	16 PE’s on FPGA chips to accelerate RNAalifold RNA alg.	2D systolic array design implemented on a Virtex-II 6000 FPGAs	16 PE’s on FPGA chips to accelerate Zuker RNA alg.
Parallel Improvements	Speed-up factor of 1.6× on execution time	Achieving a factor of ×17 on Speed-up	Achieving results in a shorter amount of time Avg. *O*(*n*^4^) speed-up for both	A factor of 12.2 × Speed-up over RNAalifold (*ViennaRNA-1.6.5*)	Achieving Speed-up up to 39× over a recent x86-family CPU	Speed-up of more than 14× over the *ViennaRNA-1.6.5*

**Table 3:** Comparison parallel algorithms for RNA secondary structure prediction.

**Parallel paradigm**	**RNA existing algorithms**	**Original complexities**		**Major contribution of the parallelization method**	**The enhancement on complexities by using parallelization design**	
		**Execution time**	**Space requirement**		**Speed-up time**	**Space requirement**
RNA prediction algorithms on multicore Parallelization “*GTfold*”	*mfold^29^,^46^* and *RNAfold*^14^	*O*(*n*^4^)	*O*(*n*^2^)	*GTfold*^15^ combine with enhancement for RNA prediction algorithms (*mfold & RNAfold*) on CPU multicore Parallelization	*O*(*n*^3^) with a factor of 1.6× on execution time	–
Accelerating RNA secondary structure algorithms on GPU	Unafold package “*hybrid-ss-min*”^48^	*O*(*n*^3^)	*O*(*n*^2^)	Adapting parallel function on *hybrid-ss-min* for RNA prediction and re-implementing on GPU	A factor of 17× on speed-up time	–
Parallel RNA predictions alg. on	*Pknots-RE*^25^	*O*(*n*^6^)	*O*(*n*^4^)	*compPknots:*^17^ a parallel framework by using combination of both existing methods (*Pknots-RE, Pknots-RG*) and running both alongside for prediction RNA structure with more accuracy and shorter time	*O*(*n*^4^)	*Avg. O*(*n*^2^)
Beowulf cluster «*Master–Slave*»	*Pknots-RG*^34^	*O*(*n*^4^)	*O*(*n*^2^)			
Parallelizing RNA secondary structure algorithms on FPGA chips	RNAalifold alg.^35^	*O*(*m × n*^4^*+ n*^3^)	*O*(*n*^2^)	A systolic array structure using fine-grained parallel on FPGAs^18^ to accelerate RNAalifold algorithm	A factor of 12× on speed-up time	–
Parallelizing Nussinov RNA structural algorithms on FPGA co-processors	Nussinov’s alg.^12^	*O*(*n*^3^)	*O*(*n*^2^)	A parallel systolic arrays on FPGA^19^ co-processors for accelerating Nussinov’s RNA algorithm	A factor of 39× on speed-up time	–
Accelerating Zuker’s algorithm for RNA structural by Parallel fine-grained on FPGA	Zuker’s algorithm^13^	*O*(*n*^4^)	*O*(*n*^2^)	A parallel systolic arrays on FPGA^20^ co-processors for accelerating Zuker’s RNA algorithm	Up to factor of 14× speed-up comparing with *ViennaPackage*	–
